# Plant Virus Nanoparticles for Anti-cancer Therapy

**DOI:** 10.3389/fbioe.2021.642794

**Published:** 2021-12-15

**Authors:** Srividhya Venkataraman, Paul Apka, Erum Shoeb, Uzma Badar, Kathleen Hefferon

**Affiliations:** ^1^ Department of Cell and Systems Biology, University of Toronto, Toronto, ON, Canada; ^2^ Theranostics and Drug Discovery Research Group, Faculty of Pharmaceutical Sciences, University of Nigeria, Nsukka, Nigeria; ^3^ Department of Pharmaceutics, Faculty of Pharmaceutical Sciences, University of Nigeria, Nsukka, Nigeria; ^4^ Department of Genetics, University of Karachi, Karachi, Pakistan

**Keywords:** nanoparticles, plant virus-like particles, therapeutics, imaging, cancer 2

## Abstract

Plant virus nanoparticles (VNPs) are inexpensive to produce, safe, biodegradable and efficacious as treatments. The applications of r plant virus nanoparticles range from epitope carriers for vaccines to agents in cancer immunotherapy. Both VNPs and virus-like particles (VLPs) are highly immunogenic and are readily phagocytosed by antigen presenting cells (APCs), which in turn elicit antigen processing and display of pathogenic epitopes on their surfaces. Since the VLPs are composed of multiple copies of their respective capsid proteins, they present repetitive multivalent scaffolds which aid in antigen presentation. Therefore, the VLPs prove to be highly suitable platforms for delivery and presentation of antigenic epitopes, resulting in induction of more robust immune response compared to those of their soluble counterparts. Since the tumor microenvironment poses the challenge of self-antigen tolerance, VLPs are preferrable platforms for delivery and display of self-antigens as well as otherwise weakly immunogenic antigens. These properties, in addition to their diminutive size, enable the VLPs to deliver vaccines to the draining lymph nodes in addition to promoting APC interactions. Furthermore, many plant viral VLPs possess inherent adjuvant properties dispensing with the requirement of additional adjuvants to stimulate immune activity. Some of the highly immunogenic VLPs elicit innate immune activity, which in turn instigate adaptive immunity in tumor micro-environments. Plant viral VLPs are nontoxic, inherently stable, and capable of being mass-produced as well as being modified with antigens and drugs, therefore providing an attractive option for eliciting anti-tumor immunity. The following review explores the use of plant viruses as epitope carrying nanoparticles and as a novel tools in cancer immunotherapy.

## Introduction

Nanomedicine is an emerging area of multidisciplinary research that has already shown promise of transforming into a disruptive innovative development (Farokhzad et al., 2008). Already, there are dozens of products in clinical trials and even some on the shelf in some pharmacies across the world though users are relatively few because of the rather prohibitive price tags of these innovative products (Park, 2019). It is pertinent to state that while a lot has been proposed in terms of the anticipated efficacy of nanomedicines, opinions tend to vary when it comes to the stage of a critical cost-benefit- analysis for the availability of nanomedicines for use in the treatment of cancer and other ailments (FDA Drug Reports, 2017).

Nanomedicines includes a wide array of nanomaterials with particle size ranging from 1nm to more than 400 nm and are a remarkably diverse group of materials (Zhang et al., 2008; Zhou et al., 2012). They may be made up of entirely of a metal as in the case of Gold and Silver nanoparticles (Paviolo and Stoddart, 2017), or a combination of liquids or a ternary system composed of an assortment of several compatible materials giving rise in most cases to a multifunctional entity often possessing stimuli responsive attributes enabling it to respond to minute changes in factors such as pH and temperature variations (Moreira et al., 2016). Additionally, nanoparticles can be prepared with simple polymeric materials such as cellulose and chitosan (Steinmetz and Manchester, 2009).

Immunotherapy in cancer treatment simply refers to a strategy with the objective of galvanizing the immune system of the patient to resist the implanting of cancerous cells. There are several approaches to achieve the desired end. One approach involves the use of drugs known as “Immune checkpoint inhibitors, to block immune checkpoints (Byun et al., 2017). The checkpoints are a typical part of the immune system and serve to modulate the immune response so that it does not come as too strong. The net effect of this treatment modality is that the blocking of these checkpoints makes it possible for the immune cells to respond more strongly to cancer.

Plant virus-based nanoparticles (VNPs) have been explored as a unique class of nanocarriers for biomedical applications (Pitek et al., 2016). In addition to their ease of production and quality control maintenance, plant virus VNPs offer a logical alternative to synthetic nanoparticles as they are inexpensive to produce, nontoxic and biodegradable (Rybickie, 2020). Plant virus nanoparticles have been further improved for their performance in terms of stimuli-responsivity (Brun, Gomez, and Suh 2017).

Plant virus nanoparticles tend to be either rod shaped, such as Tobacco mosaic virus (TMV) and Potato virus X (PVX), or icosahedral shaped, such as Cowpea mosaic virus (CPMV). Different shaped viruses respond differently as nanoparticles *in vivo*. Tobacco mosaic virus can assemble into VLPs without requiring its RNA genome carry a drug payload on the surface or to a limited extent, within the inner channel of the nanoparticle. Potato virus X, cannot self-assemble in the absence of its RNA genome, and thus can only carry a payload on the outer surface. Cowpea mosaic virus can be made to self-assemble into empty virus like particles in the absence of its RNA genome and can thus carry a payload both inside and outside of its protein shell (Sainsbury et al., 2010). In this review, we provide a series of examples to discuss how plant virus architecture contributes to their applications in cancer diagnostics and therapy (Wen et al., 2015a; Wen et al., 2015b). We discuss the architecture of plant viruses, how they came to be used as nanoparticles in various medical applications, and how they may be employed in the future as novel cancer immunotherapies (Shahgolzari et al., 2021).

### Architecture of Plant Virus Nanoparticles

Viruses are composed of outer protein shells which encapsulate the genomic material. The multiple copies of coat proteins that form the virus outer shell of viruses are collectively known as the capsid (Liu et al., 2016). Primarily, the capsid occurs in different shapes and sizes and is meant to protect the genomic material to keep viruses safe under extreme environments (Pokorski and Steinmetz 2011). The immense diversity with respect to the shape and size of plant viruses enables them to be tailored for specific applications. The structural integrity of viruses remains intact even when surface properties have been altered through chemical and genetic modification; this allows control over targeting ligands, drugs and contrast agents for imaging (Rong et al., 2011). Various chemical and genetic approaches are reported to control the virus surface properties without affecting structural integrity, and allow control on the attachment sites of drug molecules or contrast agents on the virus surface (Rong et al., 2011). Plant-virus capsid pores are also reported to be employed to encapsulate small therapeutic molecules (Zeng et al., 2013).

**FIGURE 1:**
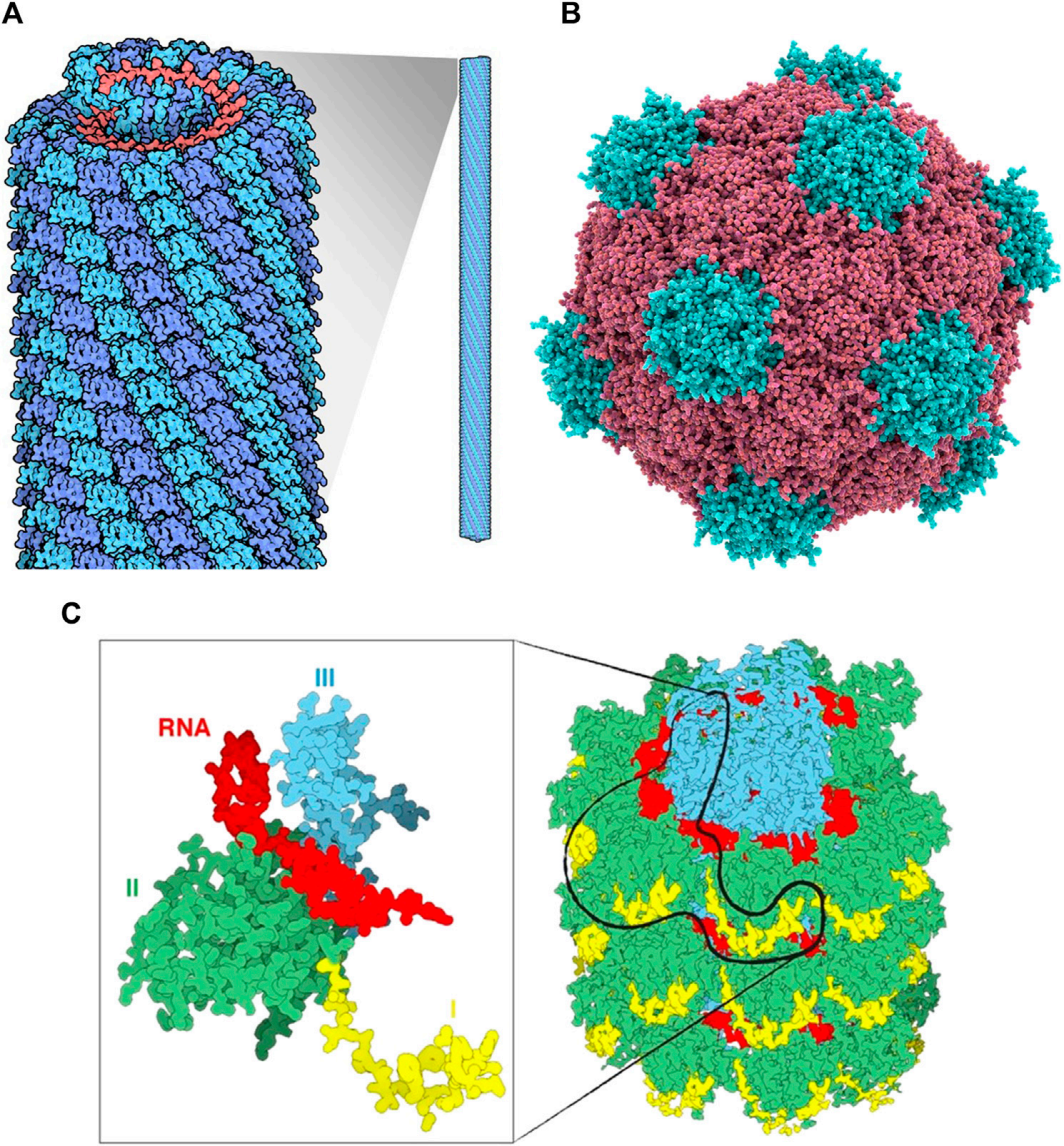
**(A)** Tobacco mosaic virus structure, RNA is in red, protein subunits in blue Source: https://pdb101.rcsb.org/motm/109. **(B)** Cowpea mosaic virus structure, Protein subunits in red and blue Source: fineartamerica. com. **(C)** An overview of a portion of the PVX virus **(right)**. The three domains of the protein are shin in yellow, green and cyan, the RNA in red. The magnification on the left displays only one single CP with a fragment of RNA.

Plant viruses have been used as virus like particles (VLPs) and virus nanoparticles (VNPs) as epitope display systems for vaccine production. VLPs are a subset of the VNPs but lack any nucleic acid genome, thus making them non-infectious. VNPs and VLPs based on plant viruses are both non-pathogenic to humans and biodegradable (Steinmetz, 2010). VNPs and VLPs are advantageous due to their ability to be generated quickly while serving as highly versatile molecular scaffolds (Young et al., 2008; Steinmetz and Evans, 2007]. Examples of plant viruses utilized as VLPs include Cowpea mosaic virus (CPMV) and Tobacco mosaic virus (TMV). An example of a plant virus utilized as a VNP is Potato virus X (PVX). These are listed in Figure 1.

Tobacco mosaic virus (TMV) is the most well-studied plant virus and was initially characterized in the 19th century. TMV can be easily produced and purified in bulk amounts and can be manipulated genetically due to its relatively simple particle structure and genome organization. The rod-shaped virus particle measures 300 nm in length and 18 nm in diameter and contains a 6.7 kb viral RNA genome that is encapsulated by 2,130 identical copies of the capsid protein assembled in a helical arrangement. At neutral pH and in the absence of RNA, the CP assembles itself into an 18 nm double disk, a 20S aggregate or nano-ring containing two layers of 17 CP molecules which can serve as a nanoscale scaffold.

Potato virus X (PVX) is a member of the family Alphaflexiviridae, genus Potexvirus, an important plant pathogen of the family Solanaceae, and specifically infects potato, tomato and tobacco (Adams et al., 2004) (Massumi et al., 2014). It has a 6.4-kb positive-stranded RNA genome (Park et al., 2008). Multiple copies of CP assemble around the genomic RNA to form the capsid. PVX can carry large payloads due to its flexible and filamentous structure, making it possible to use for pharmaceutical and imaging applications (Roder, Dickmeis, and Commandeur 2019).

The PVX particle is 515 × 14.5 nm in dimension and comprised of 1,270 subunits of CP, (Parker et al., 2002). The C-terminus of each CP subunit is located internally and the N-terminus projected externally to the assembled particle, which provides a suitable site for modification (Nemykh et al., 2008). Unlike other reported viruses, the assembly of PVX CP subunits into filamentous VLP, *in vivo* or *in vitro*, is not possible in the absence of genomic RNA. This reflects the unique connection between virus RNA and CP (Kwon et al., 2005).

The plant pathogen Cowpea mosaic virus (CPMV) belongs to the Comovirus genus. CPMV is an icosahedral shaped virus with a diameter of approximately 27 nm. It is composed of RNA-1 and RNA-2 of 6 and 3.5 kb, respectively packed in 60 copies each of Large and Small coat protein (Singh et al., 2007). CPMV is one of the most developed VNPs for biomedical and nanotechnology applications due to its ability to target specific tissues and act as an efficient drug delivery system. It is also reported to be well-adapted for the attachment of a variety of molecules to the coat protein. Five reactive lysine residues of CPMV coat protein provide sites to chemically conjugate to various compounds such as fluorescent dyes (Steinmetz, 2010). CPMV can be produced as empty virus like particles (eVLPs) Meshcheriakova et al. (2017). eVLPs are non-infectious and could be loaded with heterologous material, has increased the number of possible applications for CPMV-based particles.

### Biomedical Applications

VLPs can act as strong vaccine candidates as they simulate the conformations of native viruses, utilizing their intrinsic immunogenicity without compromising their safety. VLPs evoke effectual immune responses as they are readily internalized by antigen presenting cells (APCs) and are ideal platforms for antigen processing and epitope presentation to immune cells. VLPs are composed of multiple copies of their capsid (coat) proteins, which when assembled appear as repetitive, multivalent molecular scaffolds. As a result, the multiple copies of coat protein can facilitate the multivalent presentation of antigens fused to their surface. Therefore, VLP vaccines afford superior immunogenicity compared to antigens in their soluble states. Additionally, plant viral VLPs and VNPs possess inherent adjuvant properties dispensing with the use of additional adjuvants to evoke strong immune responses (Table 1).

**TABLE 1:** Select Examples of Plant Virus Nanoparticles and their Medical Applications.

Plant virus nanoparticle	Architecture	Use in cancer immunotherapy	Reference
**Tobacco mosaic virus (TMV)**	Rod-shaped	carrier for tumor associated carbohydrate antigens	Yin et al. (2012)
		Carrier of cancer drug phenanthriplatin in inner core of virus nanoparticle	Vernekar et al. (2018)
		*In vivo* imaging	Chung et al. (2020)
**Potato virus X (PVX)**	Rod-shaped	*In vivo* imaging	Shukla et al. (2018)
		PVX-DOX (doxorubicin) combination therapy	Lee et al. (2017)
		displays tumor necrosis factor (TNF)-related apoptosis inducing ligand (TRAIL) on the surface	Le et al. (2019)
**Cowpea Mosaic virus (CPMV)**	Icosahedral shaped	HER2 epitope displayed on virus surface	Shukla et al. (2013)
		combination therapy of CD47-blocking antibodies and CPMV nanoparticles	Wang and Steinmetz, (2019)
		slow-release aggregates comprised of polyamidoamine generation 4 dendrimers (CPMV-G4)	Czapar et al. (2018)

TMV VLPs have been utilized as epitope display systems in a variety of settings, with a first example being a polio vaccine by Haynes et al. (1986). Later, TMV was used as an epitope display vehicle for a malaria vaccine and others, including foot and mouth disease virus, human papillomavirus, norovirus, hepatitis B virus, influenza virus and human immunodeficiency virus (Turpen et al., 1995; Nooraei et al., 2021). Röder et al. (2017), were able to fuse a fluorescent protein to the C-terminus of the Tobacco mosaic virus (TMV) coat protein (CP) and also carried an N-terminal Foot-and-mouth disease virus (FMDV) 2A sequence. This enables the fusion protein to be cleaved from TMV.

Potato virus X, in contrast to TMV, has a flexuous rod shape and requires its genomic RNA to self-assemble. PVX has been used extensively as an epitope display system for vaccine research. For example, Uhde-Holzem et al. (2016), reported genetically altered PVX which displayed *Staphylococcus aureus* protein A fragments on its surface, and proved to be easily functionalized with IgG to be used in biosensing plant viruses. VLPs of Papaya mosaic virus (PapMV), of the Potexvirius family, has been engineered for use as a seasonal flu trivalent vaccine (Carignan et al., 2015; Balke and Zeltins, 2020).

CPMV has also been developed as an autonomously replicating virus vector for the expression of either peptides or polypeptides in plants (Shahgolzari et al., 2020). Examples of CPMV used as an epitope presentation system include epitopes from the outer membrane (OM) protein F of *Pseudomonas aeruginosa* which were shown to protect mice against bacterial challenge, and an epitope expressing the 30 amino acid D2 domain of the fibronectin-binding protein (FnBP) from *Staphylococcus aureus*, which has been shown to be able to protect rats against endocarditis (Liu et al., 2005).

Recently, Albakri et al. (2020), explored how CPMV particles can activate human monocytes, dendritic cells (DCs) and macrophages. Monocytes, upon incubation with CPMV *in vitro*, released the chemokines CXCL10, MIP-1α and MIP-1β into cell culture supernatants. Dendritic cells and monocyte-derived macrophages were also activated after incubation with CPMV, this activation is part of SYK signaling. Shukla et al. (2020) were able to demonstrate that CPMV outperformed many other types of VLPs to be a particularly strong immune stimulant.

The capacity for multifunctionality and multivalency makes plant nanoparticle platforms an ideal choice for theranostic applications (Beatty and Lewis 2019; Wang, 2019). Plant nanoparticles are capable of precise molecular imaging to achieve accurate cancer diagnosis and therapy (Ma et al., 2017). Delivery of imaging probes through nanostructures can improve the chances of early-stage cancer diagnosis through the use of multiple modalities to improve resolution, sensitivity, penetration, time, cost and on the top of all clinical relevance compared to the single imaging modalities (Key and Leary, 2014; Shahgolzari et al., 2020). Drug conjugated nanoparticles administered intravenously target tumors, *via* the process of enhanced permeability and retention (EPR) effect depending on the type of tumor (Hansen et al., 2015).

Molecular imaging is an emerging biomedical field which facilitates the visualization, of biological mechanisms *in vivo*. Imaging technologies can include magnetic resonance imaging (MRI), computed tomography (CT), positron emission tomography (PET) and optical imaging, which enable the monitoring of molecular and cellular processes in normal and diseased conditions in living subjects. (Chung et al., 2020). Plant VLPs can be beneficial for molecular imaging technologies than synthetic nanoparticles, due to their short half-life in circulation and their lack of side effects (Steinmetz, 2010). Furthermore, plant VLPs can be developed to carry a wide array of contrast agents and fluorescent labels, as they can be modified with antibodies, peptides and aptamers to enable enhanced targeting to specific tissues and cells.

Magnetic resonance imaging (MRI) is a promising technology for the diagnosis of disease due to its high resolution and deep contrast, however, virus-based nanoparticles have been used to increase sensitivity (Pokorski et al., 2011). TMV can act as a carrier to deliver high payloads of MRI contrast imaging agents to diseased tissues (Michael A. Bruckman et al., 2013) and fluorescent dyes for biosensing and bioimaging (Wen et al., 2015). TMV’s biological compatibility and multi-valency enables it to be a suitable carrier of *in vivo* imaging agents. For example, TMV rods have been conjugated to “BF3,” a multi-photon absorbing fluorophore which permitted mouse brain imaging over an extended duration without crossing the blood-brain barrier (Niehl et al., 2016). A bimodal contrast agent has been prepared to target integrin α2β1by loading the internal cavity of TMV nanoparticles with the complex of dysprosium (Dy3+) and the near-infrared fluorescence (NIRF) dye Cy7.5, as well as the externally conjugated with an Asp-Gly-Glu-Ala (DGEA) peptide through a linker polyethylene glycol. This nanoparticle (Dy-Cy7.5-TMV-DGEA) was stable, displayed a low cytotoxicity and achieved a high resolution when targeted to PC-3 prostate cancer cells (Hu et al., 2017).

Tobacco Mosaic Virus has been used successfully for CD imaging, targeting atherosclerosis and thrombosis by using an NIR dye as well as a targeting peptide conjugated to TMV [96]. These targeted TMV particles were able to identify atherosclerotic lesions in ApoE−/− mice upon intravenous injection, showing that TMV can be used as a platform to detect at-risk lesions.

VNPs based on PVX have been conjugated to fluorescent reporters as well [135]. As mentioned earlier, the small fluorescent iLOV protein was expressed on the surface of PVX through genetic engineering and served as a fluorescent probe which could be of potential use *in vivo* imaging. Shukla et al. (2018), reportedly produced PVX VNPs that displayed mCherry or GFP on their N-termini in *N. benthamiana* plants. Significantly, fluorescent PVX could successfully be used for *in vivo* particle tracking in an HT-29 murine model, for *in vitro* imaging of HT-29 cells, and for tracing viral infection within plants.

CPMV can also be engineered for intravital imaging (imaging living cells while they are in a multicellular organism) and improved permeability with a retention effect that improves tumor penetration (Beatty and Lewis, 2019). For *in vivo* imaging of tumors, CPMV-based VNPs have been successfully engineered to target specific tissues (Cho et al., 2014). These tumor targeting VNPs also provide biocompatible platforms for cancer therapy and intravital imaging (Beatty and Lewis, 2019).

Clinical treatment for cancer has been routinely addressed by chemotherapy (Hu et al., 2017). Regardless, the high recurrence of cancers as well as the fast clearance of anti-cancer drugs and non-targeted drug delivery necessitate the administration of maximum tolerable doses of drugs in cancer therapy, leading to increased toxicity and lower performance (Cano-Garrido et al., 2021). Therefore, drug delivery technologies that are highly targeted and promote active drug accumulation in tumors, in concert with reductions of dose requirements, could alleviate these concerns and augment treatment outcomes.

Plant virus VLPs have several attractive features that make them appropriate for targeted administration of therapeutic molecules. The anti-cancer drug doxorubicin (DOX), has been successfully delivered using VNPs and VLPs. TMV- and PVX-derived VLPs and VNPs have been successfully used to deliver DOX (Finbloom et al., 2018). In this context, helical plant VNPs such as TMV and PVX, with high aspect ratios, have proven to be of great use in effective drug delivery. VNPs have shown great promise since their cargo-RNA functions as a ruler establishing the length of the virus particle and simple adsorption of DOX on their surface was shown to be effective for reducing tumor growth (Bruckman et al., 2013; Pitek et al., 2016).

TMV can be used as a carrier of peptides with therapeutic or targeting activity against various cancers. Trastuzumab is a cancer cell inhibiting monoclonal antibody that uses the binding sites of human epidermal growth factor receptor 2 (HER2). Trastuzumab-binding peptides (TBP) are immunogenic in nature and capable of initiating production of HER2-inhibiting antibodies to seize the growth of HER2-carrying cancer cells. TMV particles displaying TBP have been created to activate this immunogenicity (Tyulkina et al., 2011). Similarly, a delivery system reported as PhenPt-TMV, with anticancer drug phenanthriplatin loaded into a hollow TMV carrier, serves as an example of stimuli responsive system, as the release of drug is induced in the presence of acidic environment (Czapar et al., 2016). Along these lines, Tian et al. (2018) demonstrated that the Transacting Activation Transduction (TAT) peptide, conjugated to the external surface of TMV, augmented internalization along with an increased ability to escape endo/lysosomal compartments. Most of these VLPS exhibited uptake by dendritic cells and macrophages and proved to be highly immunogenic. Thus, therapeutic nucleic acids can be easily delivered to immune cells during cancer treatments.

Plant virus VNPs have been used for targeted administration of platinum-based drugs against cancer. This is important as 50% of chemotherapy treatments involve the use of these platinum-derived drugs. TMV has been demonstrated to efficiently deliver Cisplatin (Franke et al., 2018) and Phenanthriplatin (Vernekar et al., 2018), both of which are platinum-based drugs. The drugs were loaded into the TMV VNP cavity using charge-driven interactions or by synthesizing stable covalent adducts. Such a TMV-based drug delivery system was proven to enable superior, targeted cytotoxicity as well as increased ease of uptake by cancer cells in in vitro systems using HepG2 and MCF-7 cancer cell lines (Liu et al., 2016).

Another anti-cancer drug, mitoxanthrone (MTO), is a topoisomerase II inhibitor and has been shown to be encapsulated by TMV (Lin and Steinmetz, 2018). VNPs exhibited superior tumor-reduction in mouse cancer models, while precluding severe cardiac outcomes that sometimes accompany direct delivery of MTO. Yet another anti-neoplastic and antimitotic drug, valine-citrulline monomethyl auristatin E (vcMMAE), was bound to the exterior of TMV VNPs which targeted non-Hodgkin’s lymphoma. Internalization into endolysosomal compartments was reported (Kernan et al., 2017), most likely accompanied by the protease-mediated release of the drug. This system was efficient in terms of cytotoxicity towards the *in vitro* Karpas 299 non-Hodgkin’s lymphoma cell line with an IC50 of 250 nM.

Helical plant virus nanoparticles (VLPs) have also been used as combination therapies to augment their immune efficacy. The PVX-DOX (doxorubicin) (Lee et al., 2017) combination was shown to be highly effective in stimulating cytokine/chemokine levels while prolonging the survival of mice in melanoma models compared to that obtained through the administration of either PVX or DOX alone.

PVX displaying TNF related apoptosis inducing ligand (TRAIL) was used to promote the recruitment and activation of death receptors *in vitro* in HCC-38 primary ductal carcinoma, BT-549 ductal carcinoma and the MDA-MB-231 breast cancer cell lines (Le et al., 2017; Röder et al., 2018). *In vivo* mouse models also demonstrated that the PVX-TRAIL formulation potently inhibited tumor growth. PVX has also been used by displaying tumor necrosis factor (TNF)-related apoptosis inducing ligand (TRAIL) on the surface of VNPs. Multivalent display of TRAIL enabled increased recruitment and stimulation of death receptors expressed on cancer cell lines (Le et al., 2019). Similarly, this formulation was shown to successfully suppress tumor growth in mice breast cancer models.

An efficient and new drug delivery system has been reported for Non-Hodgkin’s B cell lymphomas (NHL) based on PVX binding affinity towards malignant B cells. PVX loaded with monomethyl auristatin (MMAE) and administered to tissues harboring malignant B cells lead to inhibition of NHL growth in a mouse model (Shukla et al., 2020). Jobsri et al., 2015 reported a study in which PVX was conjugated to an idiotypic (Id) tumor-associated antigen (TAA) recombinant through a biotin/streptavidin linker, that elicited a 7 times higher anti-Id IgG response compared to Id alone in a mouse B-cell lymphoma model. Cytokine profiling in these mice revealed that the induction of IFN-α and IL-12, also that TLR7 was essential for viral RNA recognition.

PVX nanoparticles are increasingly being used for immunotherapy of tumor microenvironments. The monoclonal antibodies of Herceptin or Trastuzumab can be loaded onto PVX nanofilaments, which successfully induced apoptosis in breast cancer cell lines (Esfandiari et al., 2016). PVX was used as an expression vector for a mutant form of the HPV16 E7 oncoprotein, by fusing it with lichenase. This elicited protection against tumor progression in mice by inducing a robust cytotoxic T-cell response (Demurtas et al., 2013).

Steinmetz et al. (2009), found that CPMV nanoparticles could bind to vimentin, a protein found on the surface of most cells. Vimentin is upregulated during tumor progression, making it an attractive target for cancer therapy. The fact that surface vimentin expression correlated with CPMV uptake in this study demonstrated the ability of CPMV to detect invasive cancer cells.

The tumor microenvironment poses a great challenge to immune clearance by virtue of being immunosuppressive and favoring immune escape of the tumors through the inhibition of anti-tumor T-cells (Chung et al., 2020). CPMV VLP nanoparticles were shown to decrease tumor growth in murine models of lung melanomas, ovarian, colon and breast tumors (Lizotte et al., 2016; Wang et al., 2019). Mechanistically, CPMV has been shown to reprogram the tumor microenvironment by recruitment of natural killer cells and neutrophils, while enabling the transition of M2 to M1 anti-tumor macrophages. This innate immune cell population subsequently combats the tumor leading to cell lysis. Most recently, Mao et al. (2021) have deduced which TLRs are responsible for these properties.

The icosahedral shape of CPMV capsid can be loaded with precise drug cargos to target tumor and cancer cells. CPMV VNPs have also been formulated as slow-release aggregates along with polyamidoamine generation 4 dendrimers (CPMV-G4) (Czapar et al., 2018), where they were shown to be effective in combating ovarian cancer in murine models, even when provided as a single dosage.

CPMV VLPs have been attached to TAAs (tumor associated antigens) using chemical conjugation, genetic fusion and enzyme-mediated ligation techniques. For example, the human epidermal growth factor receptor 2 (HER2) epitope, when conjugated to the icosahedral CPMV, was successfully delivered to the lymphatic system with enhanced uptake and activation of APCs that led to an augmented anti-HER2 immune response. The CPMV HER2 candidate vaccine slowed tumor progression and metastasis in mouse models, enhancing survival (Shukla et al., 2013). Importantly, CPMV-HER2 stimulated a predominantly Th1 immune response while Sesbania Mosaic Virus-HER2 and CCMV-HER2 induced mostly a Th2 response in mouse models, thus proving that the nature of the epitope carrier itself plays an essential role in regulating the Th1/Th2 bias. This could be due to differences in epitope display on the surface of the VNPs as well as the capsid.

Cancer vaccines against carbohydrate antigens associated with tumors (TACAs) could be useful for diminishing tumor progression. Nevertheless, carbohydrates are weakly immunogenic and therefore, plant viruses used as carriers of these molecules could enhance the immune response to TACAs. CPMV-TACA conjugates targeting the Tn antigen (GalNAc-α-O-Ser/Thr) (Yin et al., 2012) were demonstrated to induce enhanced IgG titers, implicating heightened T-cell mediated immunity and antibody isotype switching in mouse models. IgG binding to the Tn antigens were observed in experiments wherein mice sera were added to breast cancer cell lines.

The chemotherapeutic cyclophosphamide, when used in combination with CPMV VNPs, profoundly elicited tumor cell death, releasing extracellular TAAs and stimulating immune cell invasion in addition to augmenting TAA recognition and antigen presentation (Cai et al., 2019) in mouse tumor models. CPMV VNPs have also been administered in combination with CD47-blocking antibodies (Wang and Steinmetz, 2019) which proved to have synergistic effects in combating tumor growth in murine ovarian tumor models, where it activated phagocytes, leading to stimulation of the adaptive immune response. Similar synergistic effects were observed when CPMV VNPs were used in combination with the anti-programmed cell death-1 checkpoint inhibitor (Lam et al., 2018). In addition to this, CPMV has been used successfully in promoting anti-tumor effects, when combined with radiation therapy. In this instance, CPMV was shown to enhance the recruitment of APCs, which in turn targeted the extracellular TAAs and phagocytosed them to induce a prolonged effectual immune response (Patel et al., 2018) in mice and canine models.

The CPMV-DOX conjugate was developed using eighty molecules of the chemotherapeutic drug doxorubicin (DOX), covalently bound to carboxylates at the external surface of the CPMV nanoparticle. This drug delivery vehicle was found to be more cytotoxic than free DOX when used in low concentration, however, CPMV-DOX cytotoxicity is time-delayed at higher concentrations (Aljabali et al., 2013). Cancer cells manage to resist immunotherapies owing to the immunosuppressive nature of tumors. CPMV nanoparticles have been reported as an *in situ* vaccine to stimulate an anti-tumor response and overcome local immunosuppression (Shukla et al., 2020). CPMV is also shown to be effective for ovarian cancer. The strategy for immunotherapy resulting antitumor efficacy is promising and involved the formation of aggregates of CPMV and polyamidoamine generation 4 dendrimers (CPMV-G4). Administration of CPMV-G4 effectively reduced ovarian cancer (Czapar et al., 2018). CPMV nanoparticles thus provide a therapeutic application for tumor targeting, intravital imaging and cancer therapy (Yildiz et al., 2013). Further exploration into the pharmacology of CPMV nanoparticles will further elucidate its roles in the immune response (Nkanga et al., 2021).

Patel et al. (2018), used CPMV nanoparticles in conjunction with radiotherapy to delay ovarian tumor growth in a mouse model. The treatment was able to result in an increase in tumor infiltrating lymphocytes (TILs), suggesting that this combined treatment could act as a future *in situ* tumor vaccine. Further studies by Wang and Steinmetz (2019) found that a protein known as CD47, that is widely expressed on tumor cells, prevents the action of T cells and phagocytic cells. The authors used a combination therapy of CD47-blocking antibodies and CPMV nanoparticles to act synergistically and elicit an anti-tumor immune response. The same research group also used low doses of cyclophosphamide (CPA) and CPMV nanoparticles as a combination therapy to successfully reduce mouse tumors *in vivo* (Wang and Steinmetz, 2019).

## Conclusion and Future Directions

The use of plant virus nanoparticles (VNPs) as drug delivery carriers for the treatment of infectious and chronic diseases including cancer are advantageous when compared with naked drugs (Shoeb and Hefferon, 2019; Hefferon, 2018). The most promising nanoparticle systems have been adopted from naturally occurring plant viruses. Plant viruses are ideal for drug delivery as they are safe, non-infectious and nontoxic to humans (Beatty and Lewis, 2019). Cancer cells exhibit specific antigens on the surface of tumor cells which can be identified and targeted by plant-virus based nanoparticles, thus providing a clinical application of diagnosis and therapeutics for cancer. The most promising nano-scale systems have been adopted from naturally occurring plant viruses such as Tobacco mosaic virus (TMV), Cowpea mosaic virus (CPMV), Potato virus X (PVX) and many more. Currently, these new strategies are only applied in small scale production. As these approaches undergo further development, we will witness a spectrum of possible applications in the fields of medicine and biomedical engineering.

In the future, plant virus nanoparticles will need to be developed for high throughput manufacturing. This will require the dedication of facilities that can produce many grams of plant virus nanoparticles using tens of thousands of plants (McNulty et al., 2021). Today, manufacturing facilities have been generated for plant molecular farming, and adaptations could be tailored for nanoparticles (Fausther-Bovendo and Kobiger, 2021). The regulatory pathway will require more exploration to speed the process. More research regarding how plant virus nanoparticles act upon the immune system is underway and will be needed (Mao et al., 2021). Finally, the use of plant virus chimeras or semi-synthetic plant virus nanoparticles with novel properties must be explored, as well as novel modes of administration, such as microneedle patches (Boone et al., 2020).
